# Whole brain myelin mapping using T1- and T2-weighted MR imaging data

**DOI:** 10.3389/fnhum.2014.00671

**Published:** 2014-09-02

**Authors:** Marco Ganzetti, Nicole Wenderoth, Dante Mantini

**Affiliations:** ^1^Neural Control of Movement Laboratory, Department of Heath Sciences and Technology, ETH ZurichZurich, Switzerland; ^2^Department of Experimental Psychology, University of OxfordOxford, UK; ^3^Laboratory of Movement Control and Neuroplasticity, Department of Kinesiology, KU LeuvenLeuven, Belgium

**Keywords:** brain mapping, magnetic resonance imaging, myelin enhanced contrast, brain integrity assessment, myelin mapping

## Abstract

Despite recent advancements in MR imaging, non-invasive mapping of myelin in the brain still remains an open issue. Here we attempted to provide a potential solution. Specifically, we developed a processing workflow based on T1-w and T2-w MR data to generate an optimized myelin enhanced contrast image. The workflow allows whole brain mapping using the T1-w/T2-w technique, which was originally introduced as a non-invasive method for assessing cortical myelin content. The hallmark of our approach is a retrospective calibration algorithm, applied to bias-corrected T1-w and T2-w images, that relies on image intensities outside the brain. This permits standardizing the intensity histogram of the ratio image, thereby allowing for across-subject statistical analyses. Quantitative comparisons of image histograms within and across different datasets confirmed the effectiveness of our normalization procedure. Not only did the calibrated T1-w/T2-w images exhibit a comparable intensity range, but also the shape of the intensity histograms was largely corresponding. We also assessed the reliability and specificity of the ratio image compared to other MR-based techniques, such as magnetization transfer ratio (MTR), fractional anisotropy (FA), and fluid-attenuated inversion recovery (FLAIR). With respect to these other techniques, T1-w/T2-w had consistently high values, as well as low inter-subject variability, in brain structures where myelin is most abundant. Overall, our results suggested that the T1-w/T2-w technique may be a valid tool supporting the non-invasive mapping of myelin in the brain. Therefore, it might find important applications in the study of brain development, aging and disease.

## Introduction

Myelin, the dielectric sheath surrounding neuronal axons, is an essential component for efficient brain functioning. Its main role is to facilitate long-range neuronal communication processes supporting higher-order cognitive, sensory, and motor functions. An accurate assessment of myelin *in vivo* is extremely important for a comprehensive understanding of human neurodevelopment and neurodegeneration (Staudt et al., 1994; van Buchem et al., 2001; Paus et al., 2001; Barkovich, 2005; Kizildag et al., 2005; Laule et al., 2006, 2007; Steenweg et al., 2010; Deoni et al., 2011; Glasser and Van Essen, 2011; Welker and Patton, 2012). Histopathological techniques are the gold standard for the quantitative assessment of myelin, but they can be used only post mortem (Gareau et al., 2000; Laule et al., 2006). Furthermore, histopathological investigations are typically conducted for only a limited number of regions, rather than for the whole brain. To address this problem, non-invasive imaging tools based on magnetic resonance imaging (MRI) were proposed for myelin mapping (Barkovich, 2000; Paus et al., 2001): conventional T1-weighted (T1-w) and T2-weighted (T2-w) imaging, magnetization transfer imaging, diffusion tensor imaging (DTI), fluid-attenuated inversion recovery (FLAIR), multi-component T2-relaxation imaging (MCRI), and multi-component Driven Equilibrium Single Pulse Observation of T1 and T2 (mcDESPOT).

Early MR studies used T1 and T2 relaxation times (Crooks et al., 1987; Tofts and du Boulay, 1990), which are strictly connected to changes in the interactions between water molecules and tissue macromolecules (Miot-Noirault et al., 1997), to assess the spatial distribution of myelin in the brain. The level of brightness characterizing white matter in T1-w MRI is associated with the spatial distribution of myelin-bound cholesterol such that the degree of myelin-related contrast can be inferred from T1-w images (Dobbing and Sands, 1973; Koenig, 1991). Conversely, T2 relaxation relates to proton transfers, molecular exchange and diffusion of water. Hydrophobic properties of the lipidic bilayer in myelin restrict molecular motion of protons (Miot-Noirault et al., 1997; Barkovich, 2000) and hypointensity on T2-w images reflects relatively larger myelin content. It is worth noting that T1-w and T2-w images typically provide only qualitative information on myelin distribution in the brain. Therefore, different MR techniques are preferred for clinical studies involving the direct comparison of myelin in patients and healthy controls.

Magnetization transfer imaging is the most commonly used technique to detect subtle changes in the biochemical architecture and composition of tissues (Grossman et al., 1994; Rademacher et al., 1999; van Buchem et al., 2001; Barkovich, 2005). The fundamental concept behind this modality is the exchange of magnetization between mobile protons (water) and immobile protons bound to macromolecules (non-aqueous tissue). This effect is usually measured as a magnetization transfer ratio (MTR). Despite its high sensitivity toward tissue changes and damage, MTR cannot be considered an absolute marker of myelination. In fact, a low MTR may result either from a change in myelin content or from structural changes following inflammation (Gareau et al., 2000; Laule et al., 2007).

DTI is a technique sensitive to diffusion processes of water molecules in biological tissue (Beaulieu, 2002). The kinematics of water molecules can be expressed in terms of fractional anisotropy (FA), which serves as a marker of white matter development, axonal damage, and myelin pathology. However, different studies have provided evidence that myelin is not the sole element of anisotropic water diffusion in axonal fibers (Laule et al., 2007; Madler et al., 2008). Hence, FA should be considered an indicator of fiber tract density, and only indirectly of myelin content.

Additionally, a limited number of studies have speculated about the potential of FLAIR imaging as a suitable marker of myelin maturation (Ashikaga et al., 1999; Murakami et al., 1999; Kizildag et al., 2005). FLAIR is a particular inversion-recovery sequence that can be used in brain imaging to suppress or heavily reduce the signal originated from the cerebrospinal fluid. In this regard, the detection of deep white matter lesions juxtaposed to the ventricles has shown to be extremely important in the recognition of pathological processes such as multiple sclerosis (Miller et al., 1998).

In recent years, other techniques have also been introduced, such as MCRI (MacKay et al., 1994; Whittall et al., 1997; Beaulieu et al., 1998; Gareau et al., 2000; Vidarsson et al., 2005; Laule et al., 2006; Oh et al., 2006; Madler et al., 2008) and mcDESPOT (Deoni et al., 2008, 2011). These are based on the principle that spin relaxation in a particular inhomogeneous environment may not be assumed as mono-exponential. Accordingly, they employ multiple MR pulse acquisition sequences in order to define the biophysical properties of the tissue under investigation (Laule et al., 2007). This permits to separate the signal belonging to water trapped between the myelin bilayers (myelin water) (MacKay et al., 1994) from the total MR signal, resulting in a myelin water fraction (MWF) measure. MWF is currently considered a reliable marker of myelin (Gareau et al., 2000; Laule et al., 2006). A critical challenge using both MCRI and mcDESPOT is however a perceptibly long scan time (between 10 and 25 min) (Whittall et al., 1997; Gareau et al., 2000; Oh et al., 2006; Madler et al., 2008; Deoni et al., 2011; Kitzler et al., 2012; Kolind et al., 2012), which may limit their applicability in clinical studies.

Recently, there has been a resurge of interest on T1-w and T2-w imaging for myelin mapping. Glasser and Van Essen (2011) proposed to combine T1-w and T2-w images to obtain a myelin-enhanced contrast image (Glasser et al., 2013, 2014). Compared to quantitative methods, which generally require longer acquisitions, fast scanning times make it potentially well-suited for clinical investigations. It is worth noting, however, that the T1-w/T2-w technique as described by Glasser and Van Essen (2011) is a relative measure potentially characterized by intensity scale inconsistencies across datasets, which may be present even for MR images collected with the same scanner on different days. To adress this issue, which may hamper within- and between-group statistical comparisons, the use of a calibration approach is strictly necessary. Glasser and Van Essen (2011) introduced an internal calibration based on the image histogram. Importantly, this approach may be unsuitable for studies in which myelin changes are expected as a result of a brain disease. Indeed, internal calibration attenuates global differences between patients and controls, to the point that altered myelin levels might not be detected. Also, the shape of the T1-w/T2-w image histogram may be different in patients with respect to controls, so that local changes in myelin levels in patients may be erroneously observed due to histogram equalization between patient and control groups.

Here we aimed to further develop the T1-w/T2-w technique, by tackling the problem of intensity scale inconsistencies across different datasets. We developed an analysis workflow for the calibration of T1-w/T2-w intensities in the brain using information of T1-w and T2-w intensities extracted from tissue outside the brain, thereby avoiding the problems related to the use of an internal calibration. To evaluate the effectiveness of our normalization procedure, we compared T1-w/T2-w images obtained from different MR scanners, with different sequences and acquisition parameters. Next, we examined the consistency of T1-w/T2-w across healthy individuals against other MR imaging modalities, such as MTR, FA, and FLAIR. Our results suggest that T1-w/T2-w ratio method can be a reliable and relatively fast tool for non-invasive myelin imaging.

## Methods

In this section we describe the workflow for the calibration of T1-w/T2-w images, allowing for the mapping of myelin in the human brain using T1-w and T2-w MR imaging data. Next, we show the reliability and sensitivity of T1-w/T2-w ratio method as compared to alternative techniques.

### Description of the method

#### Theoretical background

**Figure 1:**
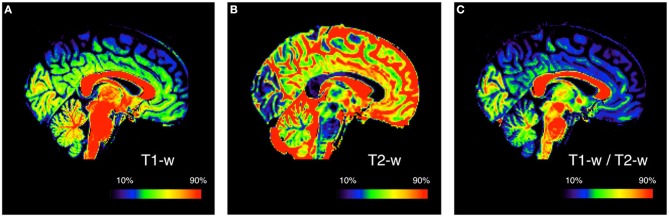
**Myelin enhanced contrast image for a representative subject**. The ratio of T1-w **(A)** to T2-w **(B)** signal intensity is calculated to obtain the T1-w/T2-w ratio image **(C)**. This is done to improve the mapping by increasing the contrast between different myelinated structures. Since conventional MRI images have arbitrary intensity scales, the three images are showed with a colormap assigned on the basis of the 10 and 90th percentile values. The subject used for this figure is Subject 30 of the KIRBY21 database.

Our method is an extension of the method originally proposed by Glasser and Van Essen (2011). They showed that, by calculating the ratio between T1-w and T2-w images of the same subject, it is possible to increase the contrast related to myelin content (Figure 1).

The myelin-enhanced contrast image obtained through this approach is however not automatically bias-free because the ratio does not attenuate (or cancel) the image bias resulting from different sensitivity profiles of the receiver coils for the two images (Belaroussi et al., 2006). Furthermore, the intensity scale of the T1-w/T2-w image is dependent on the specific instrumentation and scanning parameters used for the T1-w and T2-w images. In general terms, the T1-w/T2-w image can be modeled as follows:

(1) $$\frac{\text{T1w}}{\text{T2w}}\approx \frac{\alpha_{1}*s_{1}*x}{\alpha_{2}*s_{2}*\left(\frac{1}{x}\right)}=\frac{\alpha_{1}*s_{1}}{\alpha_{2}*s_{2}}x^{2}=\beta x^{2}$$

where the myelin content is represented by *x*, the sensitivity profiles are denoted by *s*_1_ and *s*_2_ for the T1-w and T2-w images respectively, and α_1_ and α_2_ are scaling factors. Accordingly, T1-w/T2-w intensity depends on the combination of *s*_1_, *s*_2_, α_1_, α_2_(β in Equation 1). The aim of an offline normalization procedure is to reach the ideal configuration in which both the differences in the sensitivity profiles of T1-w and T2-w sequences become negligible (i.e., *s*_1_ → 1, *s*_2_ → 1), and the values α_1_ and α_2_ are standardized, so that the T1-w/T2-w intensity scaling is comparable across different subjects.

#### Method implementation

**Figure 2:**
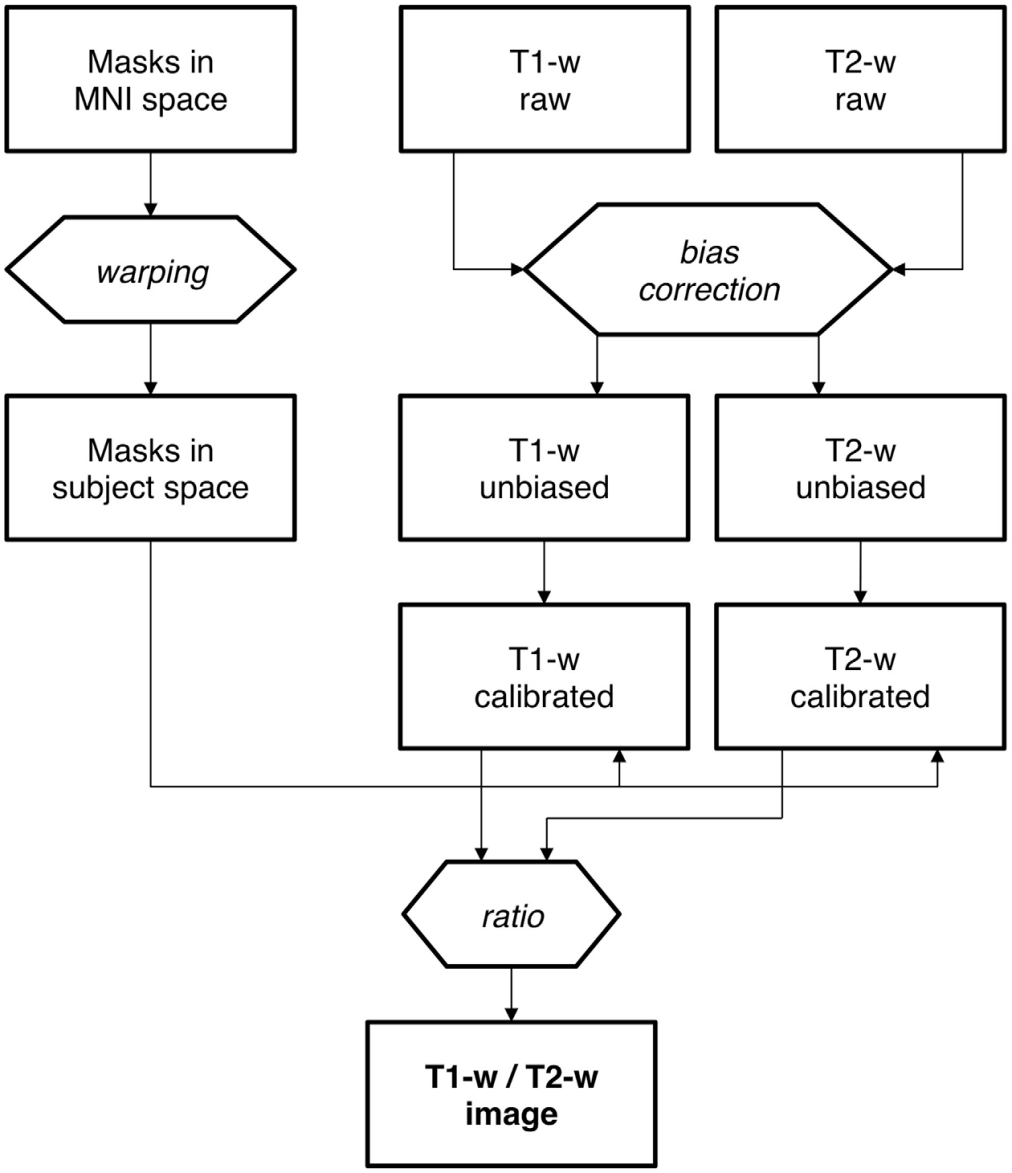
**Calibration of the T1-w/T2-w image: analysis workflow**. Workflow of T1-w/T2-w image data processing, including the warping of standard masks from MNI to subject space. The bias correction is a first, fundamental stage for both T1-w and T2-w raw images. Then each of the bias-free image undergoes the normalization process in order to accomplish a proper scaling. Finally, the T1-w/T2-w image is calculated as the ratio of calibrated T1-w and T2-w images.

**Figure 3:**
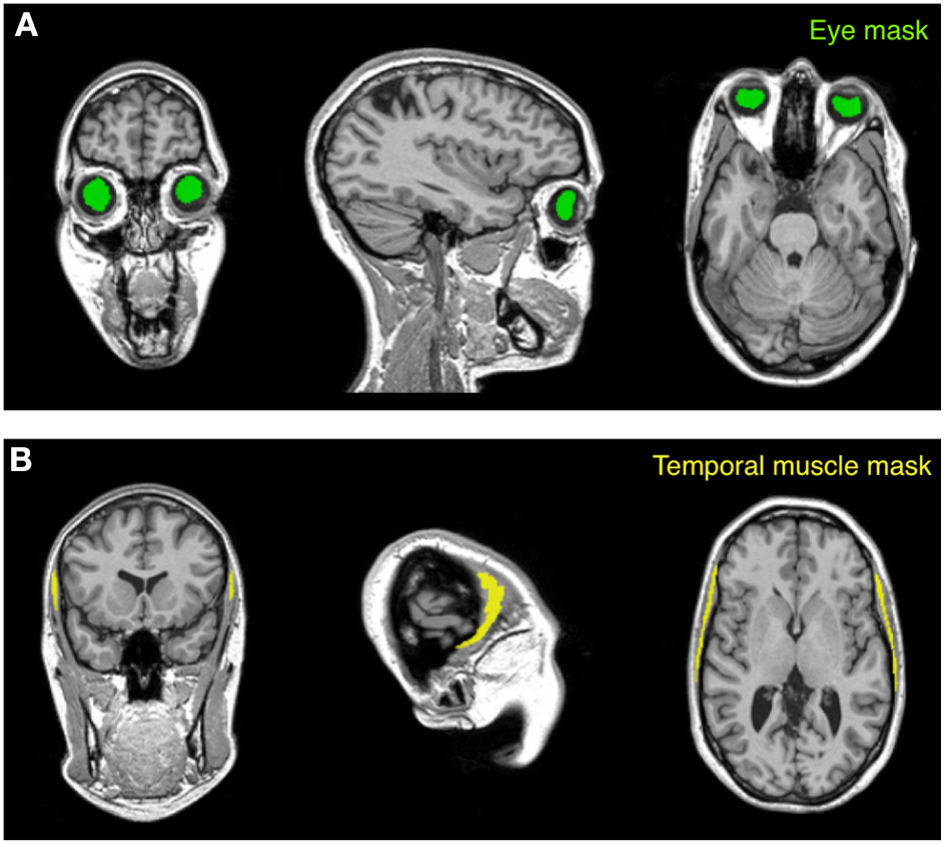
**Masks used for the calibration procedure**. The calibration algorithm is based on values extracted from two anatomical masks (eye and temporal muscle) warped to the subject space. The eye mask **(A)** is located within the vitreous humor of the eyeball and encloses the gel that fills the space between the retina and the crystalline lens. The temporal muscle mask **(B)**, which is one of the main muscles involved in the mastication process, is set on the bulk of the temporalis muscle that covers the temporal bone. The subject represented in the figure is Subject 30 of the KIRBY21 database, as in Figure 1.

***Mask creation***. Intensity standardization may be achieved with an internal scaling of intensity values, as previously proposed by Glasser and Van Essen (2011). By implementing this procedure, erroneous representation may occur in the presence of altered myelin levels. In this case, internal scaling may indeed hide substantial differences, preventing valid comparisons between controls and patients. This is the reason why we implemented an external calibration approach. The standardization of the T1-w/T2-w image was achieved through several processing steps (Figure 2), for which we used SPM8 (Wellcome Trust Centre for Neuroimaging, London, UK). As a first step, two subject-specific masks were created by warping predefined masks in the stereotaxic space of the Montreal Neurological Institute (MNI) to the individual space, using T1-w images in the two spaces to calculate the necessary spatial transformation (Ashburner and Friston, 1997, 1999). To ensure the effectiveness of this step, the masks should contain voxels outside the brain and should span image regions with relatively high regional homogeneity. Furthermore, one of them should contain relatively low values on the T1-w image and high values on the T2-w image, and the other mask should have reversed characteristics. We implemented this specification by selecting two masks covering the eyeballs and the temporal muscles, respectively (Figure 3). These were defined directly in MNI space by segmenting and thresholding the ICBM152 template images (http://www.bic.mni.mcgill.ca/ServicesAtlases/ICBM152NLin2009).

***Bias correction***. In parallel to the creation of subject-specific masks, the original T2-w image was coregistered to the T1-w image through a rigid-body transformation (Collignon et al., 1995). Then, the T1-w and T2-w images were jointly subjected to bias correction to ensure that the sensitivity profile (*s*_1_ and *s*_2_ in Equation 1) was spatially equalized. Unlike receive field (B1−) inhomogeneities, the T1-w/T2-w ratio cannot completely correct for transmit field (B1+) inhomogeneities in intensity and contrast (Glasser and Van Essen, 2011; Glasser et al., 2014). Accordingly, instead of removing common spatial inhomogeneity by combining T1-w and T2-w images (Glasser et al., 2014), we opted for using the intensity inhomogeneity correction tool implemented in SPM8 (Ashburner and Friston, 2005; Weiskopf et al., 2011) on the two images separately. The input parameters for the intensity inhomogeneity correction algorithm, namely the smoothing and the regularization parameters, were set at their default value (equal to 60 mm and 10^−4^, respectively).

**Figure 4:**
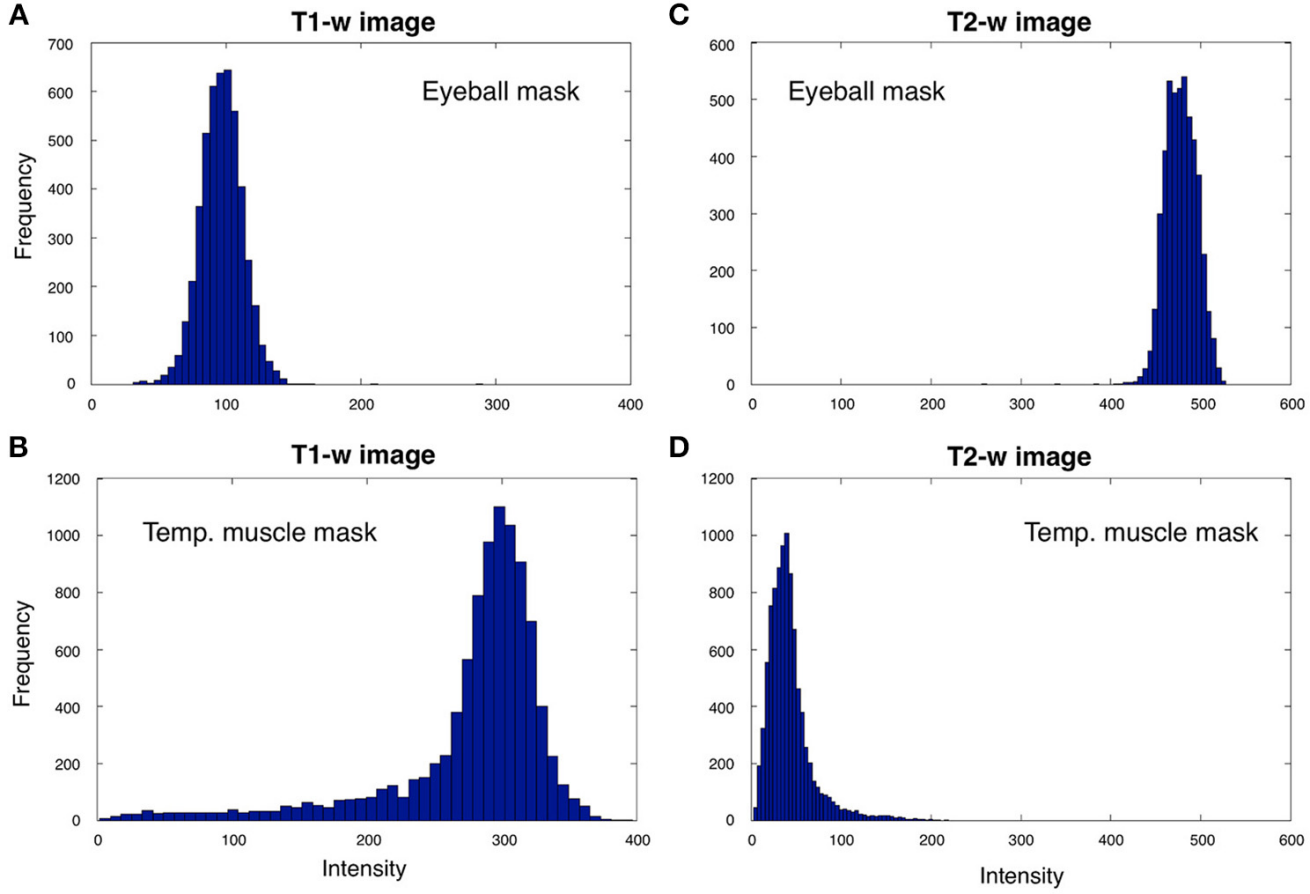
**T1-w and T2-w intensities for the eyeball and temporal muscle masks**. We analyzed T1-w and T2-w intensities within the eyeball and temporal muscle masks for Subject 30 of the KIRBY21 database. The eyeball mask values for the T1-w image **(A)** are always lower than the ones in the temporal muscle mask **(B)**, whereas eyeball mask values in T2-w image **(C)** are larger than the ones bounded by the temporal muscle mask **(D)**. Since the voxel intensities in the two masks generally showed distributions deviating from a Gaussian trend, we selected reference values as the distribution peak (i.e., the numerical mode) rather than the statistical mean or median.

***Intensity standardization***. After bias correction, the T1-w and T2-w images were further processed to standardize their intensity by using a linear scaling procedure. Specifically, the distribution peaks (modes) of intensities in the two masks (Figure 4) were extracted from either the unbiased T1-w or T2-w images of the single subject, indicated as *X_S_* and *Y_S_*, and were then compared with the corresponding values from the ICBM152 template image of the same modality, indicated as *X_R_* and *Y_R_*. The modes for the ICBM152 template corresponded to *X_R_* = 58.6 and *Y_R_* = 28.2 for the T1-w image, or *X_R_* = 21.1 and *Y_R_* = 99.9 for the T2-w image. The linear scaling of either the T1-w or the T2-w image was accomplished using the following formula:

(2) $$I_{C}=\left[\frac{X_{R}-Y_{R}}{X_{S}-Y_{S}}\right]*I+\left[\frac{X_{S}Y_{R}-X_{R}Y_{S}}{X_{S}-Y_{S}}\right]$$

where *I* and *I_C_* are the images before and after calibration, respectively. After calibrating T1-w and T2-w images with the formula described above, their ratio was calculated to produce the calibrated T1-w/T2-w image (see Equation 1).

### Method validation

#### Subjects and data acquisition

We used three different publicly available datasets for the method validation. Two of them were extracted from the IXI database of the Imperial College London (http://biomedic.doc.ic.ac.uk/brain-development/index.php?n=Main.Datasets), whereas the third was from the KIRBY21 database of the Kirby Research Center for Functional Brain Imaging in Baltimore (http://mri.kennedykrieger.org/databases.html). For the first two datasets we extracted T1-w and T2-w images collected in 21 healthy subjects with a 1.5T MR scanner (Gyroscan Intera, Philips Healthcare) and in 21 healthy subjects with a 3T MR scanner (Intera, Philips Healthcare), respectively. The third dataset contained T1-w, T2-w, MT, FA, and FLAIR images collected in 21 healthy subjects with another 3T MR scanner (Achieva, Philips Healthcare). It is worth noting that more than 600 subjects are available in the IXI database, but we selected only 21 of them for each scanner to ensure statistical comparability of the results with those from the KIRBY21 database. The selection was made such that subjects with a comparable age range across databases could be used in our analyses. We identified the optimal age-matching group in the IXI database after assessing each possible group of 21 subjects, generated using a permutation approach. We calculated the Mann–Whitney U-test on the ages of each IXI group using the ages of the KIRBY 21 group as reference. Finally, we determined the IXI group that provided the highest probability. Details on subject demographics and scanning parameters for the different image modalities are provided in Tables 1, 2, respectively.

**Table 1:** **Demographic data**.

	**IXI database (1.5 T)**	**IXI database (3 T)**	**KIRBY21 database (3 T)**
Total number of subjects	21	21	21
Number of female subjects	12	6	10
Age (min–max)	21–59	21–68	22–61
Age (mean ± *SD*)	31.7 ± 8.4	32.5 ± 12.1	31.7 ± 9.4

*A total of 63 healthy subjects were included in this study. Each dataset consisted of 21 subjects within a comparable age range*.

**Table 2:** **MR imaging sequence parameters**.

	**IXI 1.5 T dataset**		**IXI 3 T dataset**		**KIRBY21 3 T dataset**				
	**T1-w**	**T2-w**	**T1-w**	**T2-w**	**T1-w**	**T2-w**	**MTI**	**DTI**	**FLAIR**
TR (ms)	9.8	8178	9.6	5725	6.7	6653	64	6281	8000
TE (ms)	4.6	100	4.6	100	3.1	80	15	67	330
Inversion time (ms)	–	–	–	–	–	–	–	–	2400
Resolution X (mm)	1.2	0.94	1.2	0.94	1.2	0.83	0.83	2.2	0.55
Resolution Y (mm)	0.94	0.94	0.94	0.94	1.0	0.83	0.83	2.2	0.42
Resolution Z (mm)	0.94	1.25	0.94	1.25	1.0	1.5	1.5	2.2	0.42
Flip angle (degrees)	8	90	8	90	8	90	9	90	90

MR images collected with different sequences (T1-w, T2-w, MTI, DTI, and FLAIR) were used in this study. The main imaging parameters of each sequence are reported in the table. TR, repetition time; TE, echo time

#### Similarity of image histograms across subjects

We computed the T1-w/T2-w images, both before and after calibration, for each single dataset included in the study, and we compared them to assess the effects of the calibration procedure. We quantified the similarity of the intensity histograms for T1-w/T2-w images from the same MR scanner, as well as from different scanners. Specifically, we divided the whole range of image values into 500 bins and we normalized each histogram by dividing it by the sum over all its elements to account for the different number of brain voxels across individuals. We then estimated mean and standard deviation across histograms of different datasets in a bin-by-bin fashion, to quantify the consistency of the T1-w/T2-w values across subjects.

In addition, we conducted a quantitative analysis on the white matter, where myelin is mostly present. We used the SPM8 segmentation toolbox (Ashburner and Friston, 2005) on T1-w and T2-w images to create a white matter probability map that was thresholded at *p* > 0.5 to obtain a binary white matter mask. Hence, we estimated the numerical mode of the T1-w/T2-w values distribution in the mask, as representative for the whole brain structure. We applied this procedure to each dataset, and we analyzed the resulting values by descriptive and inferential statistics to evaluate a potential increase in across-subject reproducibility. We first checked that the values were normally distributed by means of a Lilliefors test (*p* < 0.05). Then we assessed whether the differences between databases were reduced by the calibration procedure using *t*-tests and a single-factor analysis of variance (ANOVA) on values before and after the calibration.

#### Comparison of T1-w/T2-w with MTR, FA, and FLAIR images

As an additional assessment, we also compared the T1-w/T2-w image with MTR, FA, FLAIR images of the same subjects, using the multi-modal imaging dataset of the KIRKY21 database. FLAIR image was only coregistered to the T1-w/T2-w image whereas MTR and FA values were calculated from the coregistered magnetization transfer images and DTI data, respectively.

Magnetization transfer imaging, being sensitive to the macromolecular composition of tissue, is classically used for the assessment of alterations in the myelin content (Schmierer et al., 2004). Magnetization transfer (MT) data are characterized by a pulsed sequence using a spoiled 3D gradient echo. For the data in the KIRBY21 database, MT preparation was achieved using a five-lobed, sinc-gauss shaped RF irradiation (B_1_ = 10.5 mT, duration 24 ms, and offset frequency = 1500 Hz). Also, a reference scan was obtained with the same parameters in the absence of MT preparation. We calculated the MTR image as:

(3) $$MTR=1-\frac{M_{S}}{M_{0}}$$

where *M_S_* is the image with MT preparation (in the presence of the radiofrequency irradiation) and *M_0_* is the reference image without MT preparation. Next, we used SPM8 to register the MTR image to T1-w and T2-w images of the same subject by a rigid-body transformation (Collignon et al., 1995).

DTI is sensitive to the diffusion of water through white matter bundles, and is commonly used to produce a map of FA values across the brain. Notably, since myelin surrounds and protects white matter fibers, the presence of high FA values can be considered an indirect index of large myelin content. The DTI data in the KIRBY21 database were acquired with a multi-slice, single-shot, echo-planar imaging (EPI), spin-echo sequence with fat suppression by spectral presaturation with inversion recovery and with anterior-posterior phase encoding direction. Diffusion weighting was applied along 32 directions with a *b*-value of 700 s/mm^2^ (Landman et al., 2011). We used the FSL 5.0 software (Oxford Centre for Functional MRI of the Brain, University of Oxford) for the calculation of the FA image. First, we performed a prealignment (similar to motion correction in fMRI data) to correct for head movement during the session and to reduce the effects of gradient coil eddy currents (Horsfield, 1999). We also used the alignment parameters to correct the B-matrix, so that information on diffusion weighting directions was correctly preserved (Leemans and Jones, 2009). Then, the diffusion tensor was calculated using a simple least squares fit of the tensor model to the diffusion data. From this, the FA image was calculated as follows (Basser et al., 1994; Pierpaoli and Basser, 1996):

(4) $$FA=\sqrt{\frac{3}{2}}\cdot \frac{\sqrt{{\left(\lambda_{1}-\bar{\lambda }\right)}^{2}+{\left(\lambda_{2}-\bar{\lambda }\right)}^{2}+{\left(\lambda_{3}-\bar{\lambda }\right)}^{2}}}{\sqrt{\lambda_{1}^{2}+\lambda_{2}{}^{2}+\lambda_{3}{}^{2}}}$$

where λ is the mean of the three eigenvalues λ_1_, λ_2_, λ_3_. After calculating FA across brain voxels, we corrected spatial mismatch between the FA map and the DTI geometric reference image in the KIRBY21 database using the SPM8 normalization tool. Next, we used again SPM8 to coregister the FA image to the T1-w and T2-w images of the same subject.

After T1-w/T2-w, MTR, FA, and FLAIR images were generated and were spatially aligned to each other, we transformed them to MNI space using the SPM8 normalization tool. This permitted us to perform across-subject statistical analyses. Specifically, we assessed the across-subject reproducibility of the different image modalities on specific regions of interest (ROIs), which were selected on the basis of previous myelin studies (Barkovich, 1988, 2000; Whittall et al., 1997; Kizildag et al., 2005; Leppert et al., 2009; Welker and Patton, 2012). A first group was composed by ROIs in the white matter and with putatively high myelin content: anterior corona radiata (ACR), superior corona radiata (SCR), pontine crossing tract (PCT), anterior limb of internal capsule (ALIC), genu of corpus callosum (GCC), splenium of corpus callosum (SCC). These ROIs were defined using the stereotaxic white matter atlas of the Laboratory of Brain Anatomical MRI, John Hopkins University School of Medicine, Baltimore, MD, USA (http://cmrm.med.jhmi.edu). A second group of ROIs included the putamen (PUT), caudate nucleus (CAU), and thalamus (THA), which are all structures with relatively low myelin content. These control ROIs were defined using the ICBM Deep Nuclei probabilistic atlas provided by the International Consortium for Brain Mapping (http://www.loni.usc.edu/ICBM).

Since we sought to compare different kinds of images that are putatively characterized by different image intensities and contrasts, we evaluated the image intensity in a single ROI against the average intensity in the whole brain, by using a two-tailed paired *t*-test. Specifically, we used the following formula:

(5) $$t_{ROI}=\sqrt{n-1}*\frac{mean(\Delta_{ROI})}{sd(\Delta_{ROI})}$$

where Δ_*ROI*_ = [*I_ROI_* − *I_BRAIN_*] is the vector with the differences between ROI intensity and full-brain mean intensity across subjects, and *n* the number of subjects. The resulting t-score reflects how much the ROI intensity differs from the mean value calculated across the brain, taking between-subject variability into account. Determining t-scores for different image modalities (T1-w/T2-w, MTR, FA, FLAIR) allowed us to assess their reliability across individuals, as well as consistency across different ROIs. Additionally, we generated a t-score map from the T1-w/T2-w data by applying the same formula in Equation 5 to each voxel rather than to a single ROI. This t-score map was thresholded at *p* < 0.05, FDR-corrected for multiple comparisons (Genovese et al., 2002), highlighting brain regions with significantly larger T1-w/T2-w values than the average across the brain.

## Results

**Figure 5:**
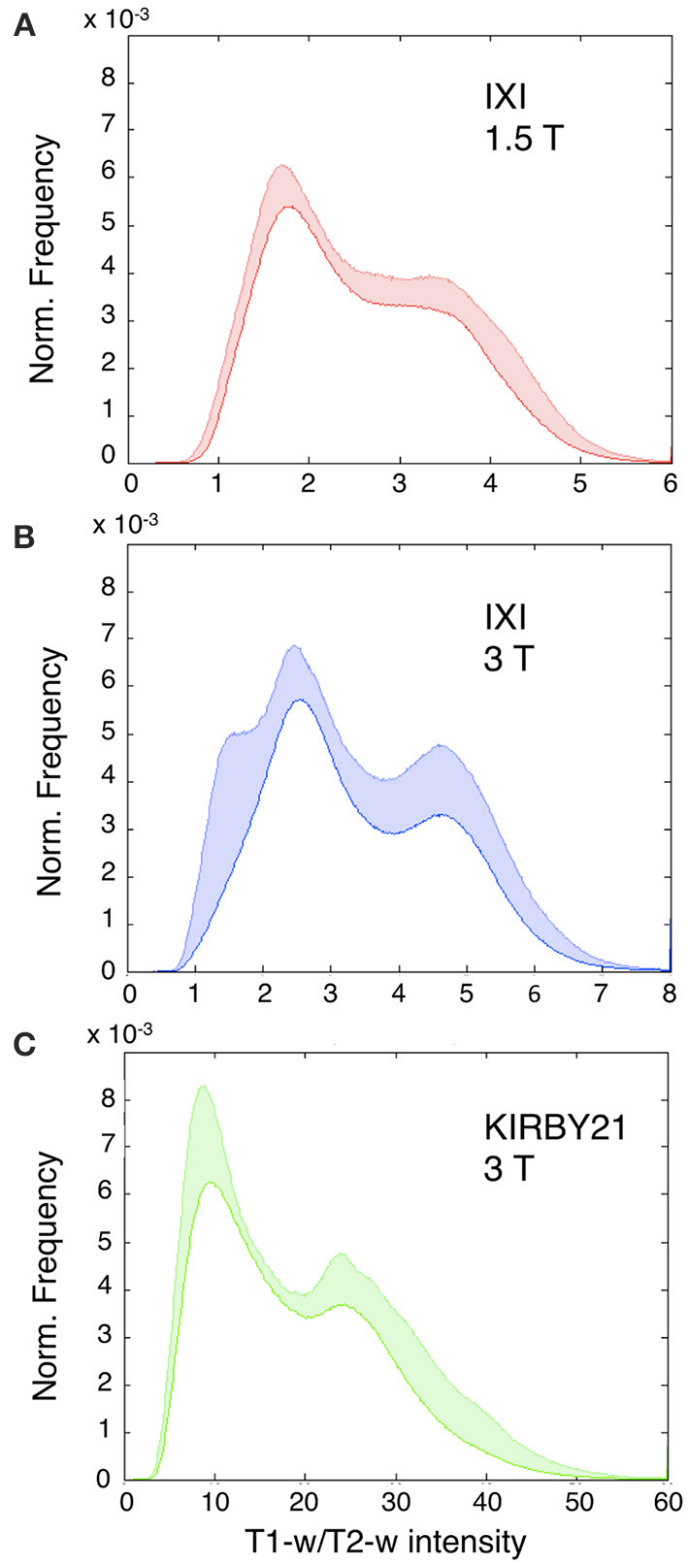
**Histograms of T1-w/T2-w image intensities before calibration**. Mean T1-w/T2-w histograms (with standard deviation in colored shade) are presented for the IXI 1.5 T **(A)**, IXI 3 T **(B)**, and KIRBY21 3 T **(C)** datasets. The three datasets have inconsistent T1-w/T2-w intensity values. **(A)** IXI 1.5 T dataset shows a relatively large inter-subject reproducibility with the smallest extent of standard deviation. **(B)** IXI 3 T dataset displays a similar trend with an increased standard deviation in correspondence to the gray matter peak. **(C)** KIRBY21 3 T dataset exhibits the greatest inter-subject variability especially in the right tail of the histogram.

As an initial analysis, we calculated T1-w/T2-w images for each dataset included in the study, using the simple ratio of unprocessed T1-w and T2-w images (as shown in Figure 1). We evaluated the variability in image histograms across datasets when no calibration procedure was applied (Figure 5). As expected, we observed that the range of intensities was largely inconsistent across the three datasets, and was especially different between the KIRBY21 dataset (Figure 5C) and the IXI datasets (Figures 5A,B). The inter-subject variability, expressed by the standard error calculated bin-by-bin across histograms, was also uneven among the three datasets. These results suggested that, although the T1-w/T2-w image can permit to map myelin distribution in an individual brain, an intensity calibration is necessary to enable meaningful comparisons across datasets.

**Figure 6:**
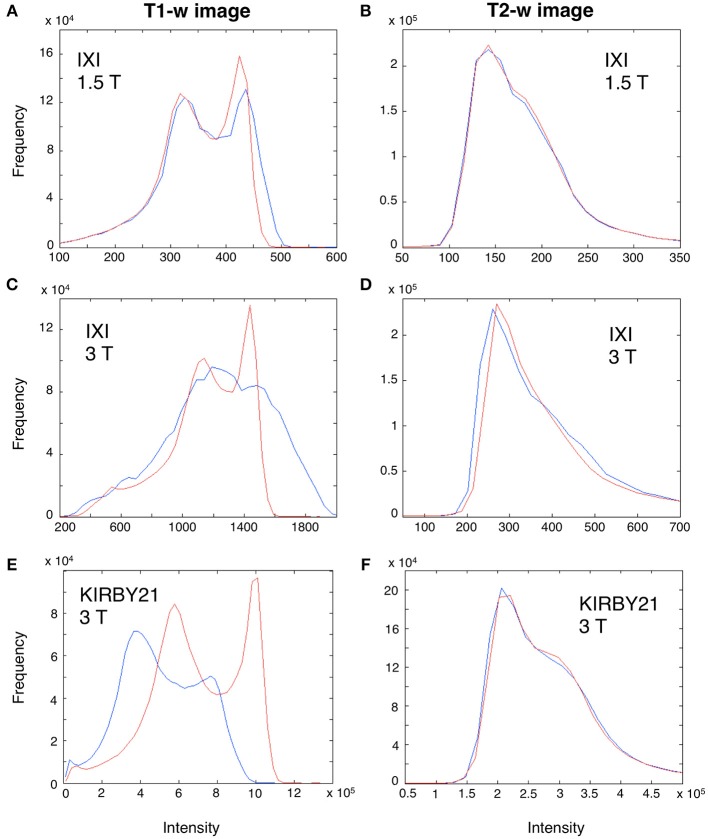
**Effect of bias correction on T1-w and T2-w images**. A modality-dependent bias correction procedure was performed for both T1-w and T2-w. To illustrate the relative results, we analyzed the T1-w **(A,C,E)** and T2-w histogram **(B,D,F)** of three representative subjects from the IXI 1.5 T (Subject 002), IXI 3 T (Subject 093) and KIRBY21 (Subject 30) databases, respectively. Specifically, we compared the histograms before (blue line) and after bias-correction (red line). Before correction, the T1-w image of IXI 1.5 T dataset **(A)** was less biased than the IXI 3 T **(C)** and the KIRBY21 3 T **(E)**, with an average correlation of *r* = 0.73. After bias correction, this correlation increased to *r* = 0.89. Conversely, in the T2-w image minor changes were observed, with an average correlation between histograms being *r* = 0.96 and *r* = 0.97 before and after bias correction, respectively.

Before using our workflow to standardize the T1-w/T2-w, we first evaluated how the bias affected T1-w and T2-w images separately, and to what extent the bias correction procedure improved the similarity of images belonging to different datasets. Visual inspection of the data suggested that T1-w images were more affected than T2-w images by the spatial bias, and in particular the latter in 3T datasets had larger magnitude than that in the 1.5 dataset (Figure 6). Importantly, we found that the image histograms of the T1-w images were variable across datasets, and the bias correction procedure strongly reduced this variability (Figures 6A,C,E). In turn, no major change in the image histogram was produced for the T2-w images (Figures 6B,D,F). Overall, this analysis suggests that bias correction step, independently implemented on T1-w and T2-w, can potentially improve the reproducibility of T1-w/T2-w histograms.

**Figure 7:**
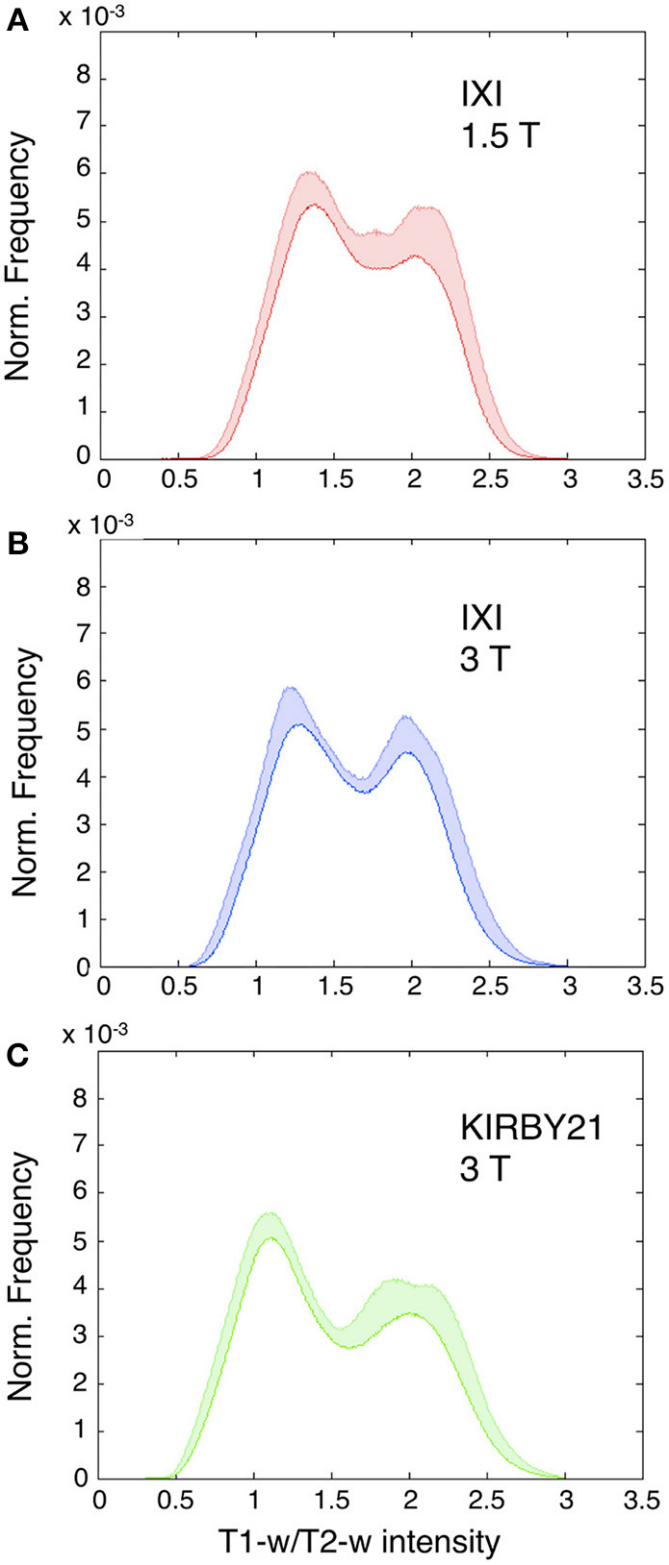
**Histograms of intensities in calibrated T1-w/T2-w images**. Mean T1-w/T2-w histograms (with standard deviation in colored shade) are presented for the IXI 1.5 T **(A)**, IXI 3 T **(B)**, and KIRBY21 3 T **(C)** datasets. In agreement with the scaling algorithm, the calibrated T1-w/T2-w images exhibit comparable intensity scales with a reduced inter-subject variability for each dataset. Note the normalized frequency on the vertical scale obtained as the ratio of each subject-specific histogram to the total area beneath the curve.

Next, we applied the linear calibration algorithm to the bias-corrected T1-w and T2-w images (see Figure 2), and we calculated again T1-w/T2-w images for the three datasets. Notably, the calibrated T1-w/T2-w image histograms (Figure 7) exhibited comparable intensity scales and reduced inter-subject variability within each dataset. A quantitative analysis conducted on white matter voxels revealed that T1-w/T2-w image values were significantly more aligned after calibration. Specifically, a clear decrease of inter-subject variability for all three datasets confirmed the effectiveness of our approach (Table 3). After checking that the values were normally distributed (Lilliefors test, *p* < 0.05), we also performed a single-factor ANOVA in order to assess the correspondence of the T1-w/T2-w means in the three datasets. The differences were significant before [*F*_(2, 62)_ = 568.48, *p* < 0.001], but not after calibration [*F*_(2, 62)_ = 1.54, *p* = 0.2236], further suggesting that the calibration procedure improved the reproducibility of the T1-w/T2-w values across datasets.

**Table 3:** **T1-w/T2-w reliability assessment**.

	**IXI 1.5 T dataset**		**IXI 3 T dataset**		**KIRBY21 3 T dataset**	
	**Before calibration**	**After calibration**	**Before calibration**	**After calibration**	**Before calibration**	**After calibration**
Mean	3.44	2.11	4.52	2.04	28.49	2.07
*SD*	0.31	0.15	0.82	0.10	4.63	0.14
t-score	50.0	64.9	25.3	91.1	28.2	69.1

*We quantitatively analyzed T1-w/T2-w values in the white matter, in order to assess the potentially increased reproducibility across subject and scanners. Specifically, we compared the numerical mode of the T1-w/T2-w values in the white matter before and after retrospective calibration, by means of descriptive (mean and standard deviation) and inferential statistics (t-score)*.

**Figure 8:**
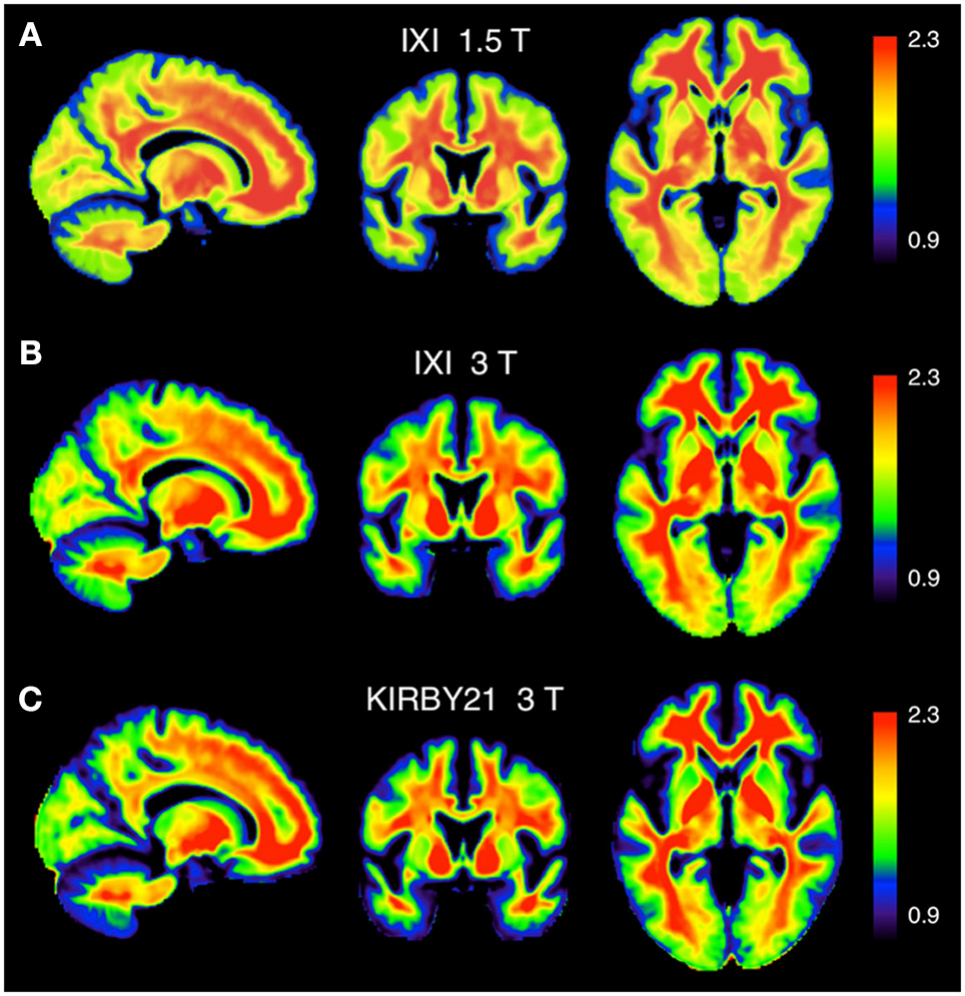
**Calibrated T1-w/T2-w images: comparison between different datasets**. The group-level T1-w/T2-w image for the IXI 1.5 T **(A)**, IXI 3 T **(B)**, and KIRBY21 3 T **(C)** datasets is shown in sagittal, coronal and axial sections. We use here a common intensity range across datasets (T1-w/T2-w values between 0.9 and 2.3).

To assess the spatial distribution of the T1-w/T2-w values, we also calculated the average T1-w/T2-w image for each of the three datasets. We set a common colormap scale to highlight potential differences among intensities in the three resulting images. Even so, we observed a very consistent spatial pattern among datasets, with no outlying features (Figure 8).

**Figure 9:**
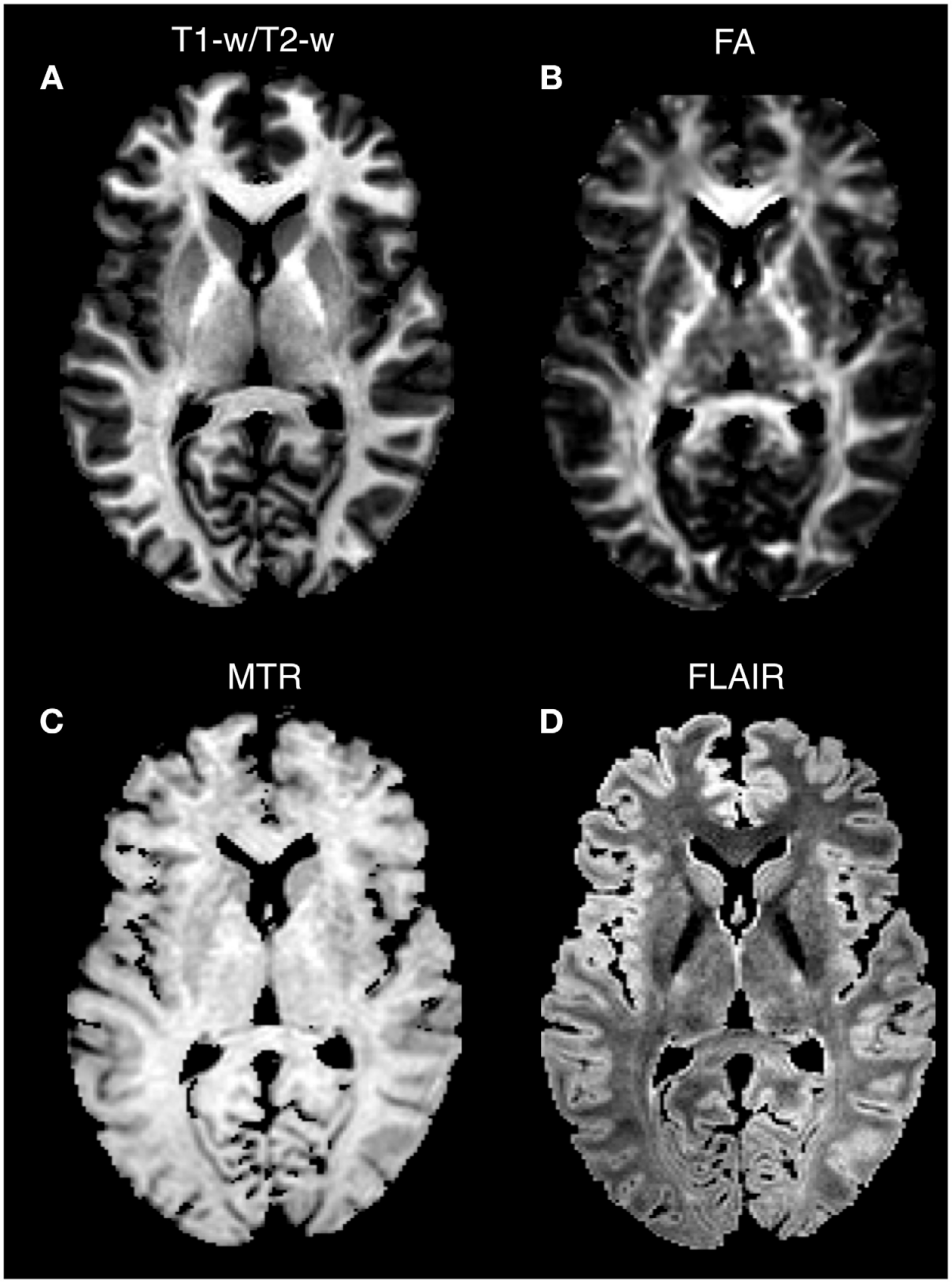
**Single-subject images: comparison between image modalities**. T1-w/T2-w **(A)**, FA **(B)**, MTR **(C)**, and FLAIR **(D)** images for Subject 30 of the KIRBY database are shown in an axial section. Since conventional MRI images have arbitrary intensity scales, the four modalities are scaled according to the 1 and 99th percentiles. Note that higher intensity values in T1-w/T2-w, FA, and MTR characterize structures with a greater degree of myelination, whereas an inverted intensity scale defines the FLAIR technique.

**Figure 10:**
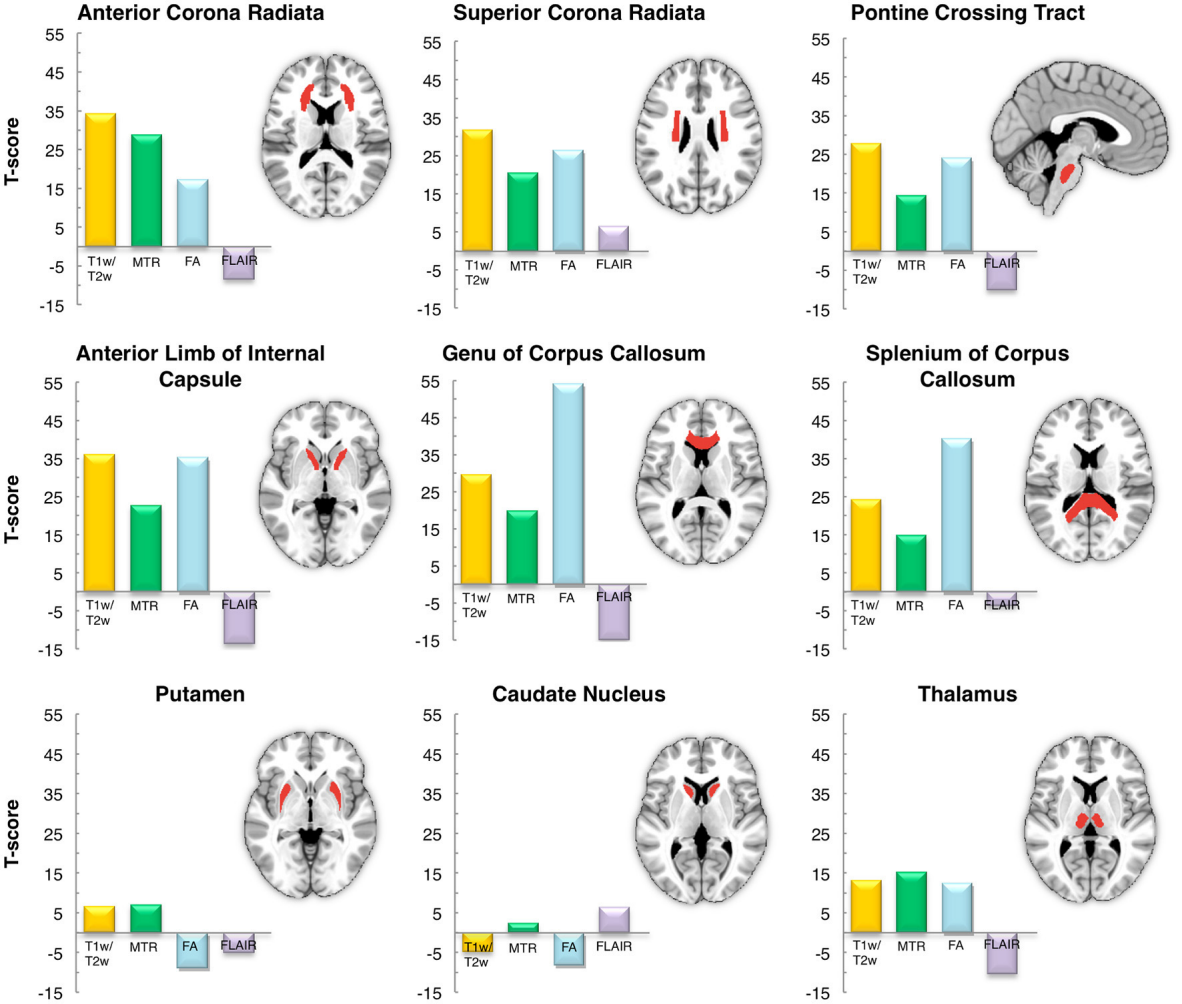
**Inter-subject reproducibility: ROI analysis**. The across-subject reproducibility of the T1-w/T2-w images as compared to MTR, FA and FLAIR were evaluated on specific ROIs. The analysis was conducted on six white matter structures and three subcortical gray matter deep nuclei with putatively high and low myelin content, respectively.

**Figure 11:**
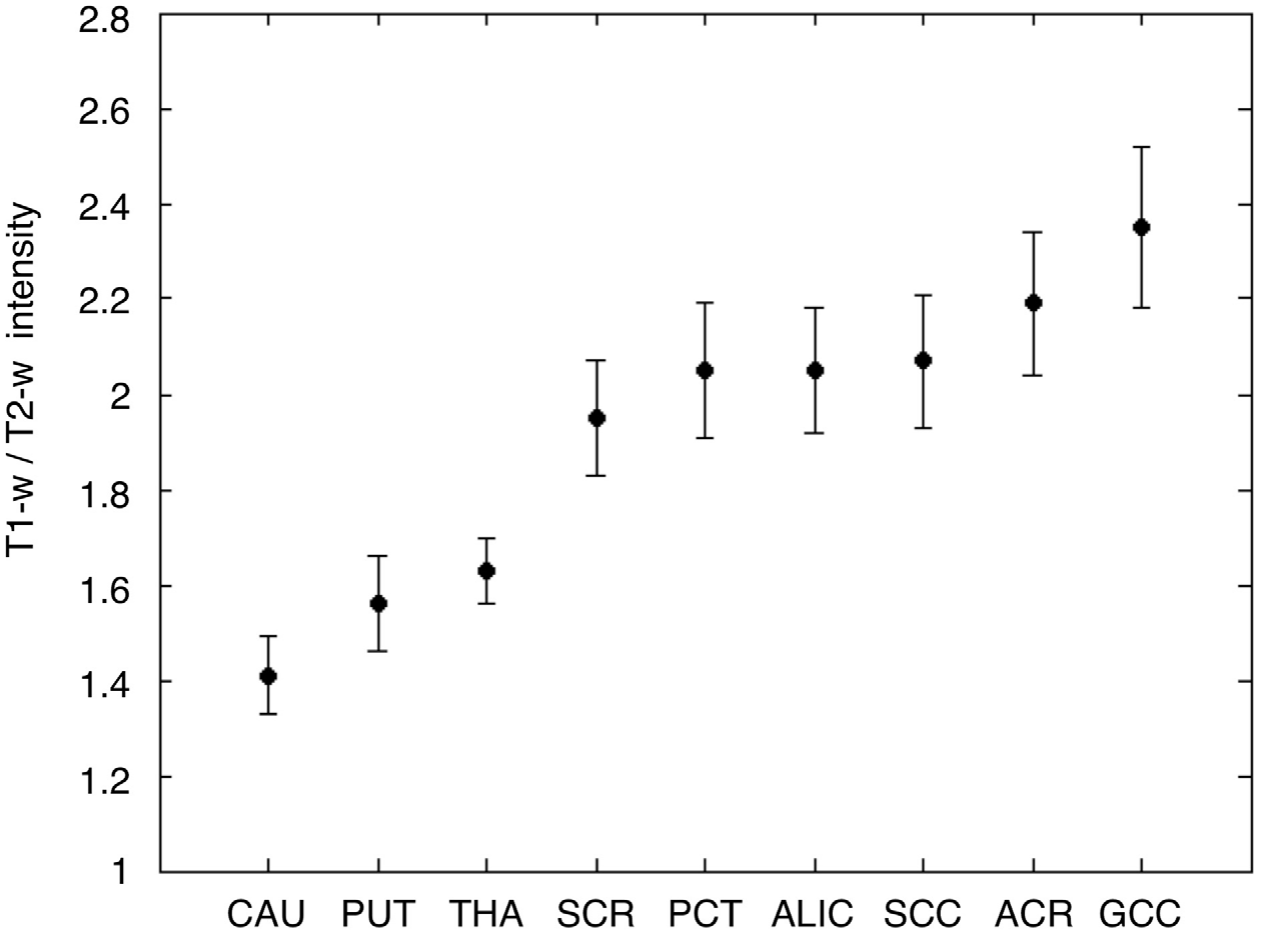
**T1-w/T2-w intensities in gray and white matter ROIs**. Average and standard deviation of T1-w/T2-w values are shown for the nine selected ROIs, three in the gray matter, and six in the white matter. As expected, T1-w/T2-w values were lower in the gray matter and in the white matter. The ROIs are labeled as follows: caudate nucleus (CAU), putamen (PUT), thalamus (THA), superior corona radiata (SCR), pontine crossing tract (PCT), anterior limb of internal capsule (ALIC), splenium of corpus callosum (SCC), anterior corona radiata (ACR), and genu of corpus callosum (GCC).

**Figure 12:**
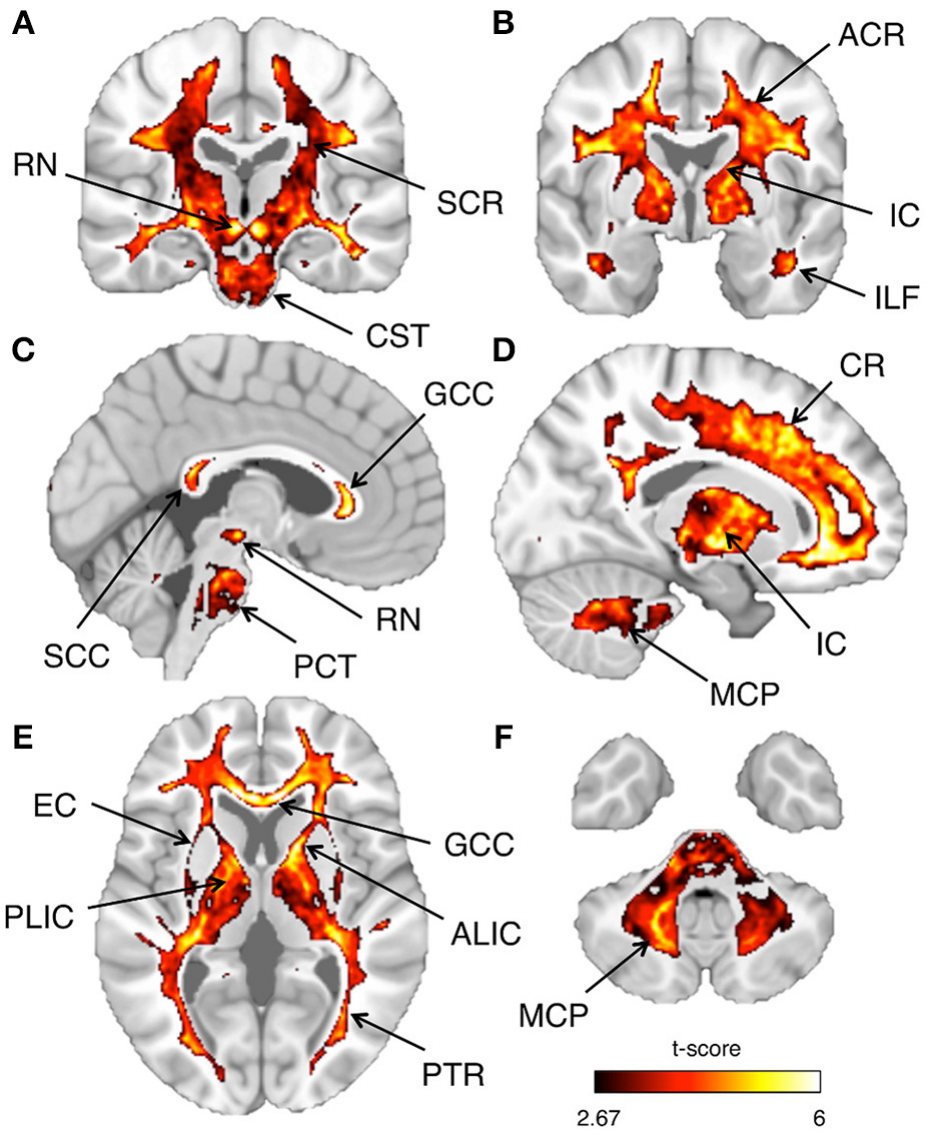
**Inter-subject reproducibility: whole-brain analysis**. The across-subject reproducibility of the T1-w/T2-w images were evaluated on a voxel-wise basis. The maps illustrate regions with significant t-scores (*p* < 0.05 FDR-corrected) over coronal, sagittal, or axial sections of a standard MNI template. The following sections, referring to the MNI coordinate system, are shown: *x* = 3 **(A)**, *x* = 17 **(B)**, *y* = −20 **(C)**, *y* = −2 **(D)**, *z* = 5 **(E)**, *z* = −39 **(F)**. Selected structures with significant t-score are indicated using arrows: anterior corona radiata (ACR), superior corona radiata (SCR), corona radiata (CR), anterior limb internal capsule (ALIC), posterior limb internal capsule (PLIC), internal capsule (IC), external capsule (EC), posterior thalamic radiation (PTR), genu of corpus callosum (GCC), splenium of corpus callosum (SCC), inferior longitudinal fasciculus (ILF), corticospinal tract (CST), pontine crossing tract (PCT), middle cerebellar peduncle (MCP), and red nucleus (RN).

As a last validation step, we compared the calibrated T1-w/T2-w images with other images, namely FA, MTR, and FLAIR images, obtained from the same subjects (Figure 9). By means of one-sample *t*-tests, we specifically tested the across-subject reproducibility and sensitivity of the four image modalities in detecting myelin-related signals. This statistical analysis conducted on different ROIs revealed that T1-w/T2-w had large reproducibility (indicated by large t-scores), which was mostly consistent across all selected white matter structures (Figure 10). The greatest t-score value was measured in the ALIC, which is consistent with the largest myelin concentration revealed by other studies (Whittall et al., 1997). As expected, the gray matter ROIs had lower values than white matter structures, both in terms of mean values (Figure 11) and t-scores (Figure 10). Overall, the t-scores obtained for MTR were inferior to those of T1-w/T2-w, but these two modalities showed a good similarity both in terms of white matter and deep gray matter structures. Also, we observed high FA values, comparable on average to those of T1-w/T2-w, but much more uneven across brain regions. In structures with multiple fiber crossing, e.g., the ACR, FA values were lower than those of T1-w/T2-w and MTR. Conversely, regions with the greater anisotropy, such as the genu and splenium of the corpus callosum, exhibited larger t-scores than T1-w/T2-w. With the exception of the SCR, FLAIR results had negative t-scores. This is consistent with the specific FLAIR image property, for which more myelinated areas have darker contrast then less myelinated ones. On the other hand, FLAIR images were characterized by low absolute t-score values, indicating a relatively low reliability and sensitivity for myelin mapping. To corroborate our T1-w/T2-w results on selected ROIs, we repeated the same analysis based on t-scores at the single voxel level. The resulting t-score map (Figure 12) showed the six white matter structures used in the ROI analysis, but not the gray matter ones, to have significantly larger T1-w/T2-w values than the average in the brain. We also observed additional structures to have significant t-score values, among the posterior thalamic radiation, inferior longitudinal fasciculus, corticospinal tract, middle cerebellar peduncle, and red nucleus.

## Discussion

In this study, we have optimized the T1-w/T2-w methodology for non-invasive myelin mapping such that inference can be drawn at group level. Our retrospective calibration procedure yielded consistent ranges of T1-w/T2-w intensities across datasets, and this may enable potential comparisons and meta-analyses across different studies and individuals. Moreover, our statistical analyses suggested that T1-w/T2-w may be a more sensitive tool for myelin imaging than MTR, FA, and FLAIR, and may therefore have future clinical applications.

### Methodological considerations on the T1-w/T2-w approach

The T1-w/T2-w approach was originally proposed by Glasser and Van Essen (2011), who showed how the contrast related to myelin content can be increased by performing the simple ratio between T1-w and T2-w images (Glasser et al., 2014). An important caveat of this approach is that the sensitivity profile of T1-w and T2-w images should be similar to yield a reliable T1-w/T2-w image. According to Belaroussi et al. (2006), this is an unlikely scenario, and this is also confirmed by our analyses (see Figure 6). The different image bias between the T1-w and T2-w images of the same subject might primarily depend on the fact that pulse sequences, and in particular the repetition time (TR) and the number of echoes, significantly influence the spatial uniformity of image intensities (Belaroussi et al., 2006). To address the issue of different image sensitivity in T1-w and T2-w images, we have included a bias-correction step in our analysis workflow (Figure 2). This substantially attenuates the slowly changing and smooth spatial variation in signal intensity that depends on the scanning hardware, the imaging parameters and the subject themselves (Belaroussi et al., 2006; Vovk et al., 2007), thereby leading to a more reliable intensity calibration.

From a methodological point of view, the image normalization is probably the most important step in our processing workflow for the T1-w/T2-w image. Frequently, a qualitative comparison between different images is achieved with an internal scaling of intensity values. This procedure, normally known as histogram equalization, consists of rescaling the image on the basis of the whole brain intensity distribution only. In this case, a color palette can be used for a visual evaluation of the image (Glasser and Van Essen, 2011), but no quantitative analysis across different images can be conducted. In general, a prospective approach permitting quantitative analyses on data produced by a single MR scanner is the use of a phantom-based calibration (Tofts, 1998). On the other hand, a retrospective approach would nevertheless be needed to perform quantitative multi-scanner comparisons. On grounds of these considerations, we implemented a retrospective calibration using image values from outside the brain. This involved the definition of reference T1-w and T2-w intensity values in the eye and temporal muscle masks to obtain a calibration curve. By using a linear scaling, we aimed to translate the intensity scale of a single image into a set of standardized values.

The comparison of image histograms within and across the three datasets confirmed the effectiveness of our retrospective calibration. The two IXI datasets had the same scanning parameters, but they were collected with a 1.5T and 3T MR scanners, respectively (Table 2). This may be the reason why their T1-w/T2-w images spanned a different range of values (Figure 5). Also, the KIRBY21 dataset deviated consistently from the other two, showing an altered pattern mainly on the right tail of the histogram, likely because of the different pulse sequence parameters (Table 2). As a matter of fact, variations of repetition time (TR) and echo time (TE) may yield different histogram distributions. In addition to these differences between datasets, large differences within datasets were also evident before calibration. These differences may be due to instrumentation factors, such as temperature and humidity, or by interactions with the subject's tissues. After calibration, the T1-w/T2-w histograms had comparable intensity scale and a similar standard deviation across datasets (Figure 7), suggesting that differences in intra- and between-dataset reproducibility were substantially reduced. Furthermore, the consistency of representative T1-w/T2-w values for the white matter across subjects confirmed this finding in a quantitative manner (Table 3). The observed effectiveness of the calibration procedure to standardize T1-w/T2-w values across subjects opened up the way to numerical analyses focused on the reliability of the T1-w/T2-w approach with respect to other myelin-related imaging techniques (Figure 10).

### Myelin-related information in T1-w/T2-w images

Previous studies documented that myelin is distributed unevenly between white matter and gray matter structures (Paus et al., 2001; Barkovich, 2005). Thus, we clustered these structures in two groups to assess the specificity of the T1-w/T2-w technique. The analysis that we conducted on selected ROIs showed high T1-w/T2-w scores in those white matter structures where myelin is most abundant (Barkovich, 1988; Kizildag et al., 2005; Leppert et al., 2009; Welker and Patton, 2012). In large accordance with our T1-w/T2-w results, previous studies reported a high degree of myelination for projection fibers, e.g., the internal capsule, corona radiata, and commissural fiber tracts including the genu and the splenium of the corpus callosum (Rademacher et al., 1999; Barkovich, 2000; Steenweg et al., 2010; Deoni et al., 2011). Furthermore, the T1-w/T2-w value in the ALIC was the highest among all investigated white matter structures, which is concordant with previous reports on the spatial distribution of myelin in the brain (Whittall et al., 1997). As for the gray matter structures, the thalamus exhibited higher T1-w/T2-w scores than did the putamen and the caudate nucleus, corroborating results reported in previous studies (Whittall et al., 1997; Madler et al., 2008).

Overall, the results of our ROI analysis for the T1-w/T2-w were also consistent with previous T2-multicomponent relaxation and MTR studies. For example, by using T2-multicomponent relaxation, Vidarsson et al. (2005) found the greatest values of MWF in the internal capsule, genu, and SCC. Markedly reduced myelin content was also found in the putamen (Vidarsson et al., 2005). Smith et al. (2006) reported high MTR values in correspondence of densely packed white matter regions, such as the callosal fibers and the internal capsule as compared to less densely packed structures. On the other hand, they found lower, but not negligible values in gray matter structures, such as putamen and caudate nucleus, in accordance with our findings (Smith et al., 2006). Since MTR is one of the most widely used techniques to study myelination (Schmierer et al., 2004), the correspondence that we observed in terms of t-scores between T1-w/T2-w and MTR in our study (Figure 10) may be considered as an indirect evidence for the potential effectiveness of T1-w/T2-w for quantitative myelin mapping.

A substantial difference in the ROI analysis results was found between T1-w/T2-w and FLAIR, with overall lower values for the latter modality. The FLAIR technique was previously employed for qualitative analyses on pathological processes related to myelination (Ashikaga et al., 1999; Murakami et al., 1999), but to the best of our knowledge it has not been employed in quantitative studies. Specifically, our comparative analysis showed that FLAIR images had relatively low t-scores in both white and gray matter structures. Accordingly, myelin assessment may not be considered the key hallmark of this technique.

Another important finding in our ROI analysis was that T1-w/T2-w and FA values were generally high, but T1-w/T2-w scores were substantially more uniform than FA across white matter structures. In first instance, this might be interpreted as evidence that FA is a sensitive technique to detect quantitative differences between regions. Nonetheless, closer inspection of FA t-scores across ROIs indicates that the FA variability may be partly due to the crossing fibers problem (Madler et al., 2008; Wedeen et al., 2008), which specifically affects DTI-derived measures. In line with previous studies (Barkovich, 2005; Provenzale et al., 2007), FA was indeed found to be high in structures with a highly organized fiber placement, such as the corpus callosum and the internal capsule, whereas it was relatively lower in regions where fibers with different orientation cross, as for example in a significant portion of the ACR (see Figure 10) (Assaf and Pasternak, 2008; Wedeen et al., 2008). This is in agreement with the proposal that, although myelin sheets contribute to anisotropy, other factors such as axonal membrane might substantially contribute to large FA values (Beaulieu, 2002; Huang et al., 2006).

### Potential limitations of the method

Our analyses suggested that our T1-w/T2-w workflow may be potentially useful for the myelin mapping in the human brain. Nonetheless some potential limitations of our study should be acknowledged. A first limitation is that only a limited number of datasets were used in this study. Images collected with very different pulse sequences may generate inconsistent results in terms of image contrast. Accordingly, the effectiveness of this approach for meta-analyses should be assessed in future studies, by examining a broader range of datasets. Secondly, our calibration procedure strongly depends on the accuracy of the calibration masks, which is in turn influenced by the effectiveness of the spatial warping from the MNI space to the subject space. To address this issue, we have extracted calibration values using the numerical modes of the mask intensity distributions. This is likely to mitigate the problem of the mask definition. It is also worth noting that our T1-w/T2-w calibration relies on the assumption that across-subject variability in the tissue selected through the masks is negligible compared to the potential differences that can be observed across the brain of different subjects. In this regard, our analysis on healthy subjects yielded largely similar T1-w/T2-w image histograms, thereby suggesting that such an assumption may generally hold. Furthermore, we utilized external calibration using eye and temporal muscle masks in alternative to internal calibration because the latter type of scaling could hide quantitative differences between healthy groups and those with altered myelin. However, diseases that cause altered myelin levels also might affect the external calibration points, e.g., temporal muscle wasting/composition change. In this case, differences between healthy and pathological groups would be underestimated or overestimated using an external calibration approach. Another aspect to be considered is that the T1-w/T2-w image in diseased individuals may be altered not only due to demyelination, but also to edema, inflammation, iron accumulation, or atrophy. This needs to be further investigated by using information from histological samples. Finally, we could not compare the T1-w/T2-w technique with all existing MR techniques for myelin mapping. Future studies should be conducted, for instance, to quantitatively compare T1-w/T2-w and mcDESPOT modalities.

## Conclusion

In this study, we implemented a new analysis workflow for the standardization of T1-w/T2-w images, thereby enabling the use of the T1-w/T2-w technique for a non-invasive mapping of myelin at group level. Our statistical analyses on selected ROIs suggested that T1-w/T2-w may permit extracting reliable information on myelin distribution, with potentially larger sensitivity than other techniques such as MTR, FA, and FLAIR. Future work is warranted to examine the potential utility of the T1-w/T2-w technique for myelination studies on development and aging, as well as for comparative investigations between healthy individuals and patients with neurological and psychiatric disease.

### Conflict of interest statement

The Reviewer Nela Cicmil declares that, despite being affiliated to the same institution as authors Dante Mantini and Marco Ganzetti, the review process was handled objectively and no conflict of interest exists. The authors declare that the research was conducted in the absence of any commercial or financial relationships that could be construed as a potential conflict of interest.
